# Supplementary figures and images for: SARS-CoV-2 spike protein induces the cytokine release syndrome by stimulating T cells to produce more IL-2
Source: Front Immunol. 2024 Sep 18;15:1444643. doi: 10.3389/fimmu.2024.1444643 (PMC11445618; doi:10.3389/fimmu.2024.1444643)

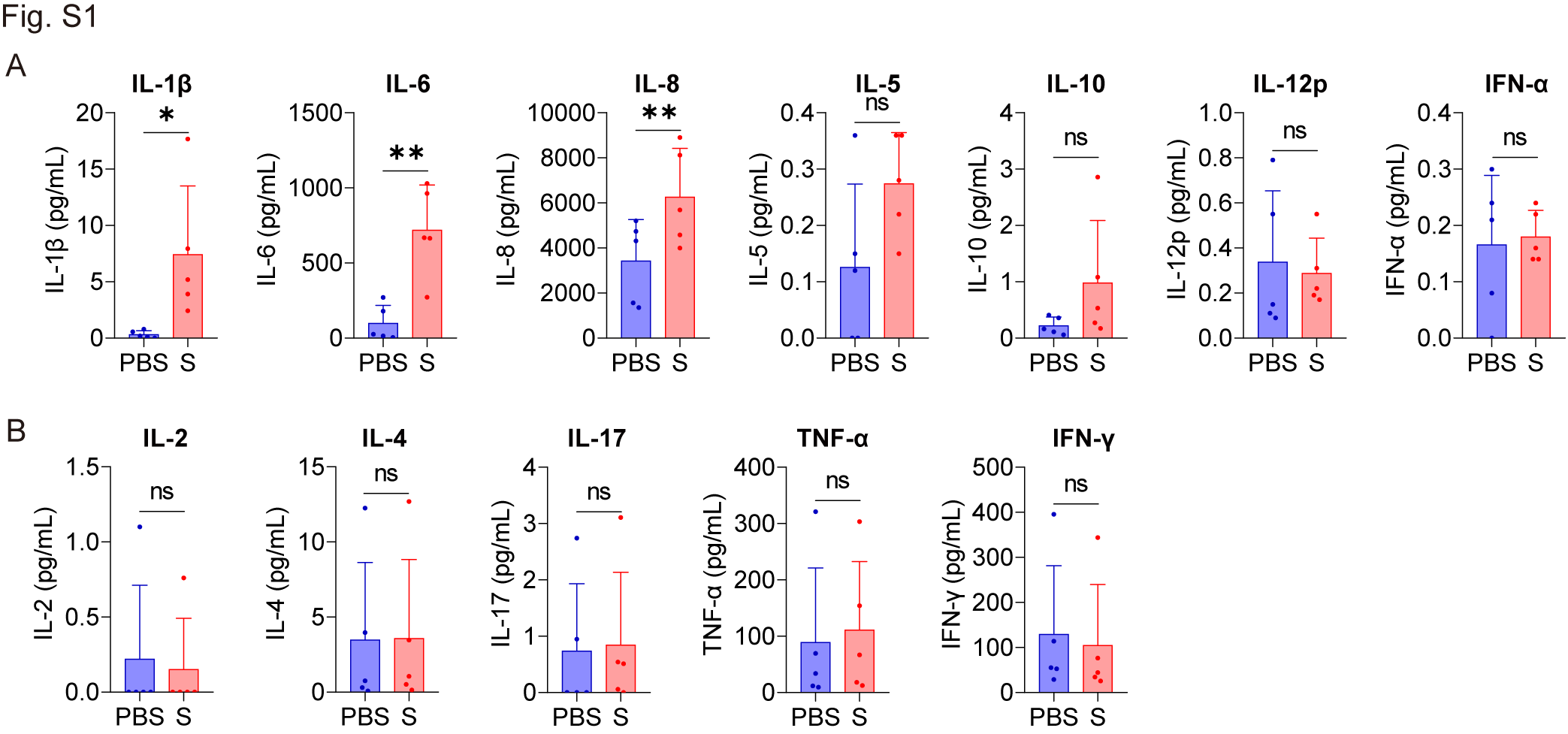

Supplement: Supplementary Figure S1 — Spike protein stimulates PBMCs to secrete IL-1β, IL-6, and IL-8. Quantifying concentrations of cytokines by CBA in the supernatants of PBMCs stimulated by spike protein for 16 hrs (n = 5 biological replicates). S, spike protein. Data are presented as mean ± SD. ns, not significant, *p < 0.05, and **p < 0.01 as analyzed by paired Student’s t-test (A) or Mann-Whitney U test (B). [file Image1.tif]

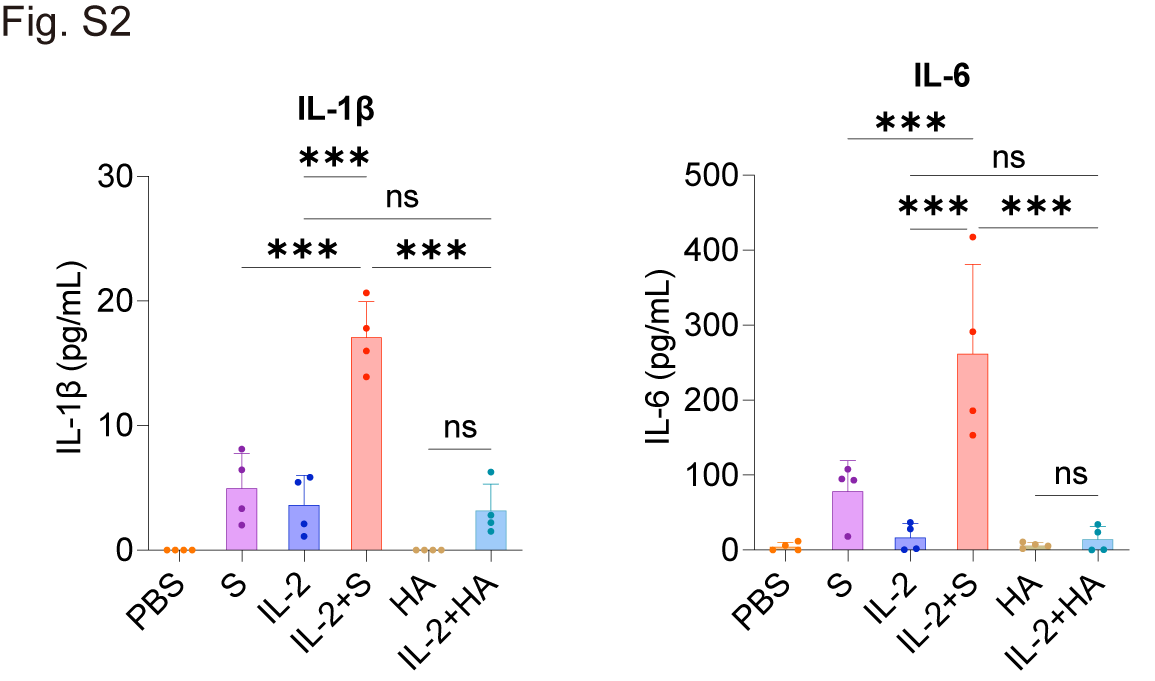

Supplement: Supplementary Figure S2 — IL-2 cannot cooperate with hemagglutinin protein to stimulate PBMCs secreting IL-1β and IL-6. Quantifying concentrations of cytokines by CBA in the supernatants of PBMCs treated with PBS, spike protein, IL-2, IL-2 combined with spike protein (S), hemagglutinin protein (HA), or IL-2 combined with HA for 16 hrs (n = 5 biological replicates). Data are presented as mean ± SD. ns, not significant and ***p < 0.001 as analyzed by one-way ANOVA. [file Image2.tif]

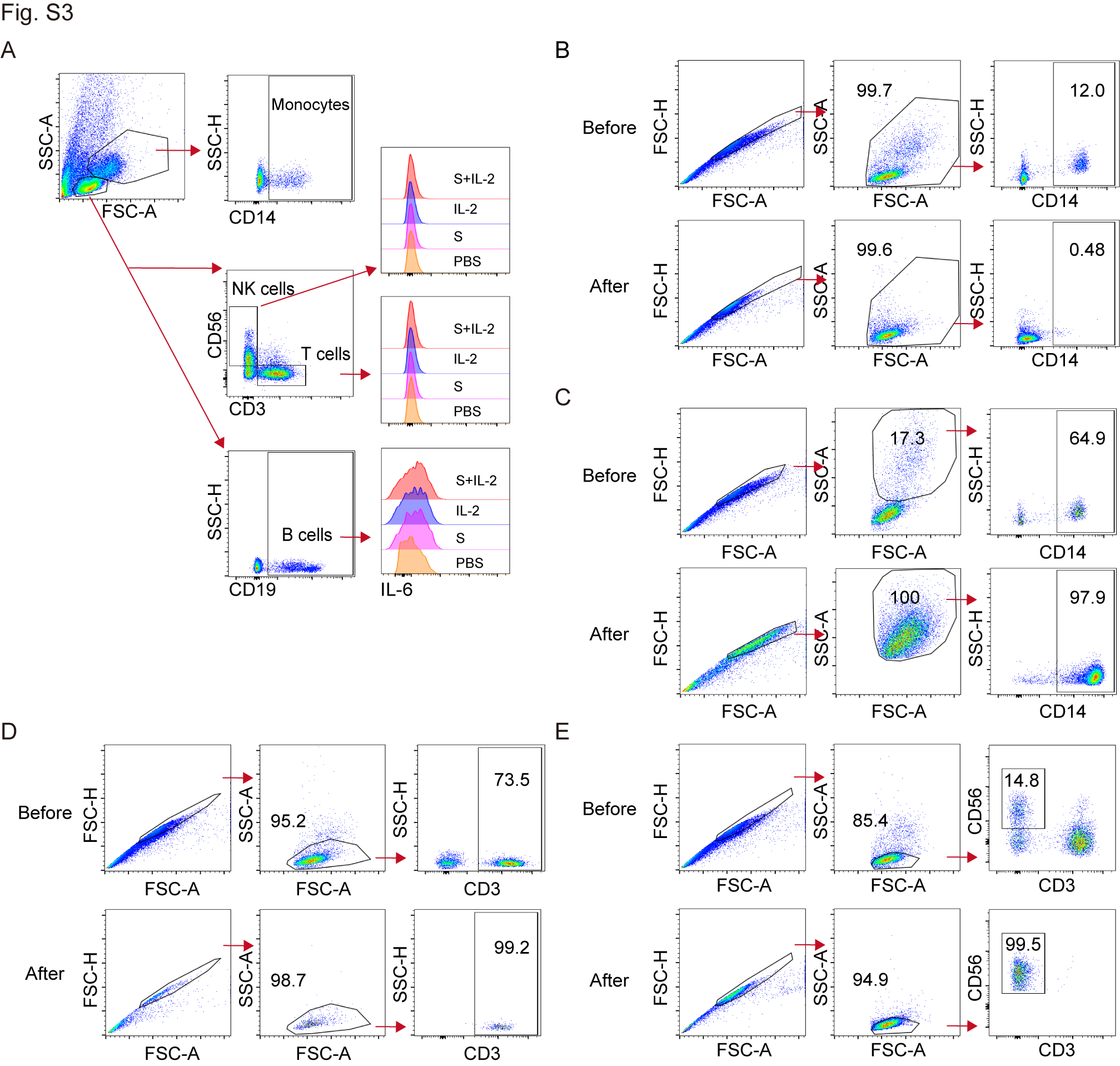

Supplement: Supplementary Figure S3 — Analysis of IL-6 expression in different immune cells in PBMCs. (A) Representative intracellular staining analysis of the expression of IL-6 using flow cytometry in monocytes, NK cells, T cells, and B cells. The analysis was carried out on PBMCs that were stimulated with PBS, spike protein, IL-2, or spike protein combined with IL-2 for 16 hours. (B) Compare PBMCs before and after removing monocytes. (C) Compare the purity of monocytes before and after sorting from PBMCs. (D) Compare the purity of T cells before and after sorting from PBMCs. (E) Compare the purity of NK cells before and after sorting from PBMCs. [file Image3.tif]

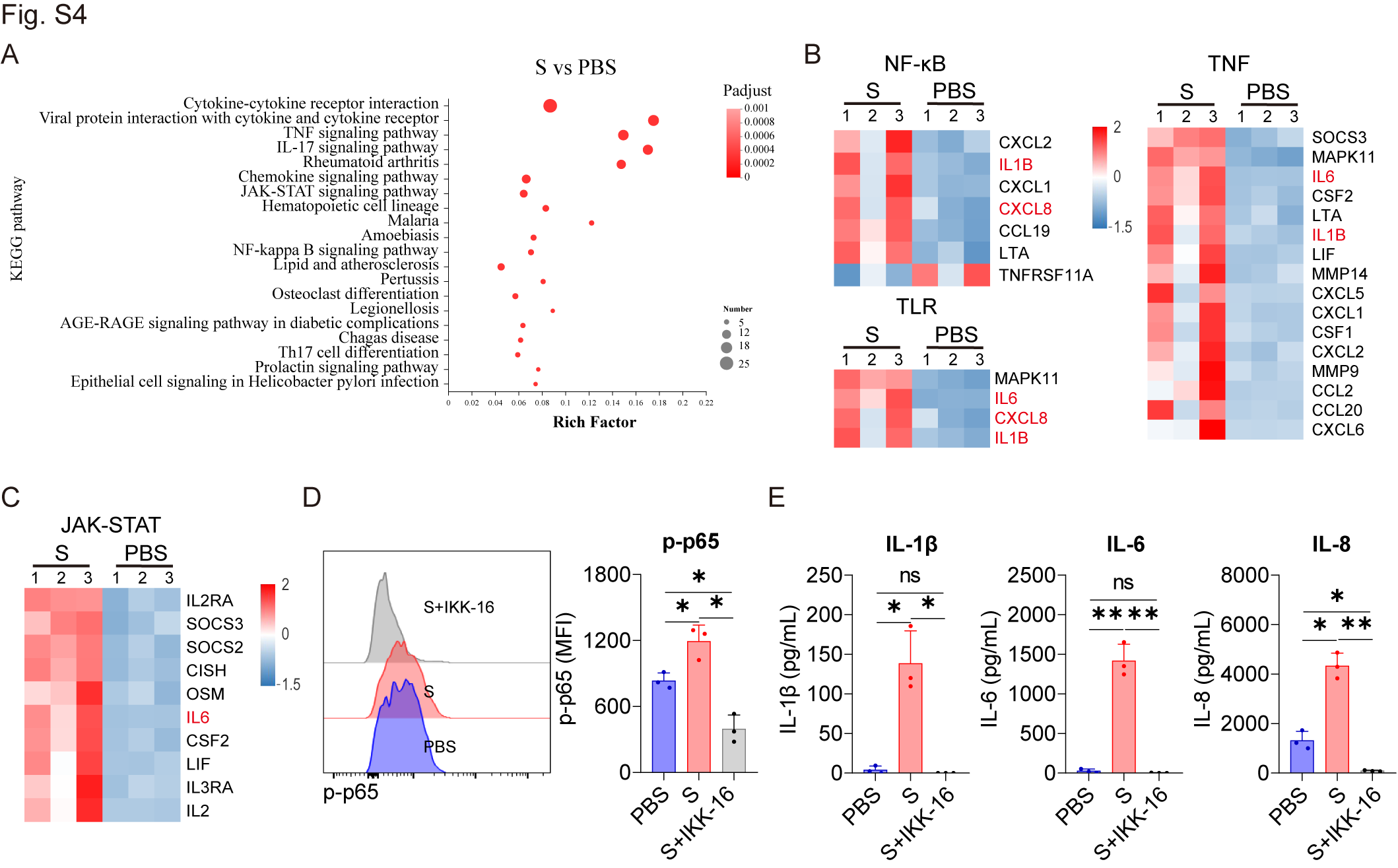

Supplement: Supplementary Figure S4 — Spike protein activates NF-κB to facilitate monocyte transcription of IL-1β, IL-6, and IL-8. (A) Bubble plot showing the enrichment of KEGG pathways based on the transcriptomic analysis of PBMCs treated with spike protein or PBS for 16 hrs. (B, C) Heat maps showing the differentially expressed genes in the NF-κB signaling pathway, TNF-α signaling pathway, and JAK-STAT signaling pathway in PBMCs treated with spike protein or PBS for 16 hrs (n = 3 biological replicates). (D) Representative intracellular staining analysis (left) and quantification (right) of the expression of p-p65 by flow cytometry in monocytes of PBMCs stimulated with PBS, spike protein, or spike protein combined with IKK-16 (n = 3 biological replicates). (E) Quantifying concentrations of IL-1β, IL-6, and IL-8 by CBA in the supernatants of PBMCs stimulated with PBS, spike protein, or spike protein combined with IKK-16 for 16 hrs (n = 3 biological replicates). S, spike protein. Data are presented as mean ± SD. ns, not significant, *p < 0.05 and **p < 0.01 as analyzed by one-way ANOVA (D, E). [file Image4.tif]

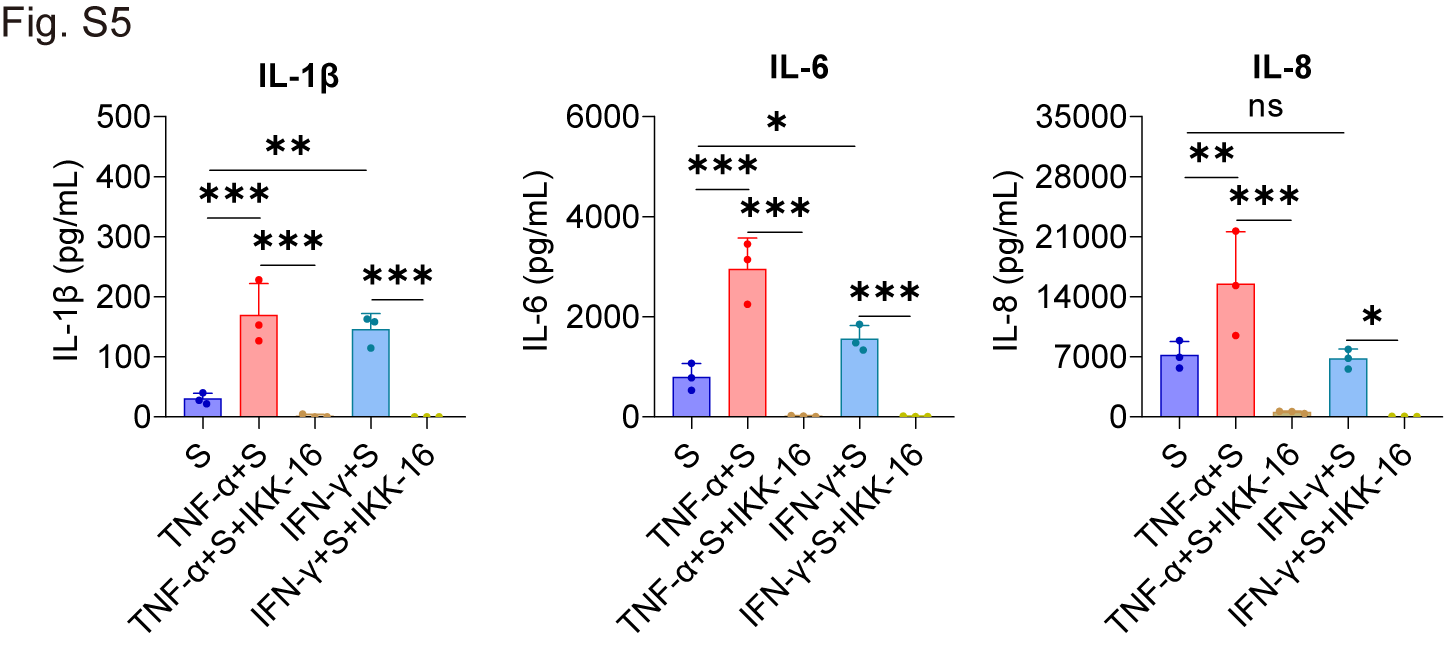

Supplement: Supplementary Figure S5 — Inhibition of NF-κB reduces the secretion of IL-1β, IL-6, and IL-8 in PBMCs stimulated by spike protein together with TNF-α or IFN-γ. Quantifying concentrations of IL-1β, IL-6, and IL-8 by CBA in the supernatants of PBMCs stimulated with spike protein, spike protein combined with TNF-α or IFN-γ, and spike protein combined with TNF-α or IFN-γ together with IKK-16 for 16 hrs (n = 3 biological replicates). S, spike protein. Data are presented as mean ± SD. ns, not significant, *p < 0.05, **p < 0.01, and ***p < 0.001 as analyzed by one-way ANOVA. [file Image5.tif]

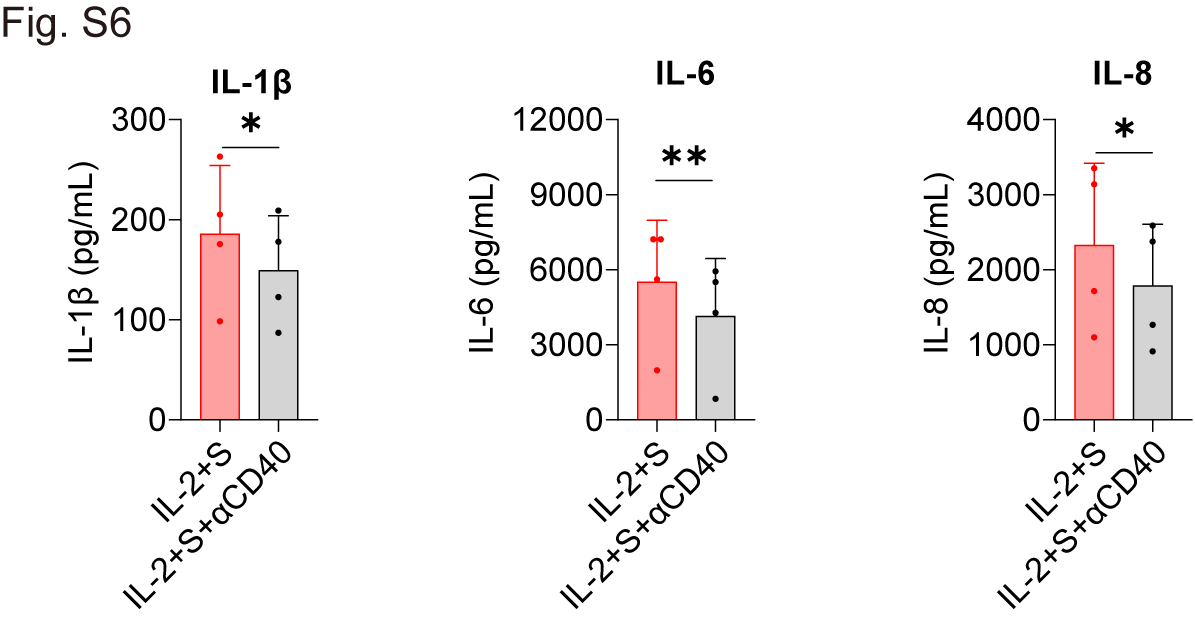

Supplement: Supplementary Figure S6 — Reduction in the secretion of IL-1β, IL-6, and IL-8 in PBMCs stimulated with IL-2 and spike protein upon blocking CD40. Quantifying concentrations of IL-1β, IL-6, and IL-8 by CBA in the supernatants of PBMCs stimulated with spike protein combined with IL-2 with/without CD40 blocking antibody for 16 hrs (n = 4 biological replicates). S, spike protein. Data are presented as mean ± SD. *p < 0.05, and **p < 0.01 as analyzed by paired Student’s t-test. [file Image6.tif]

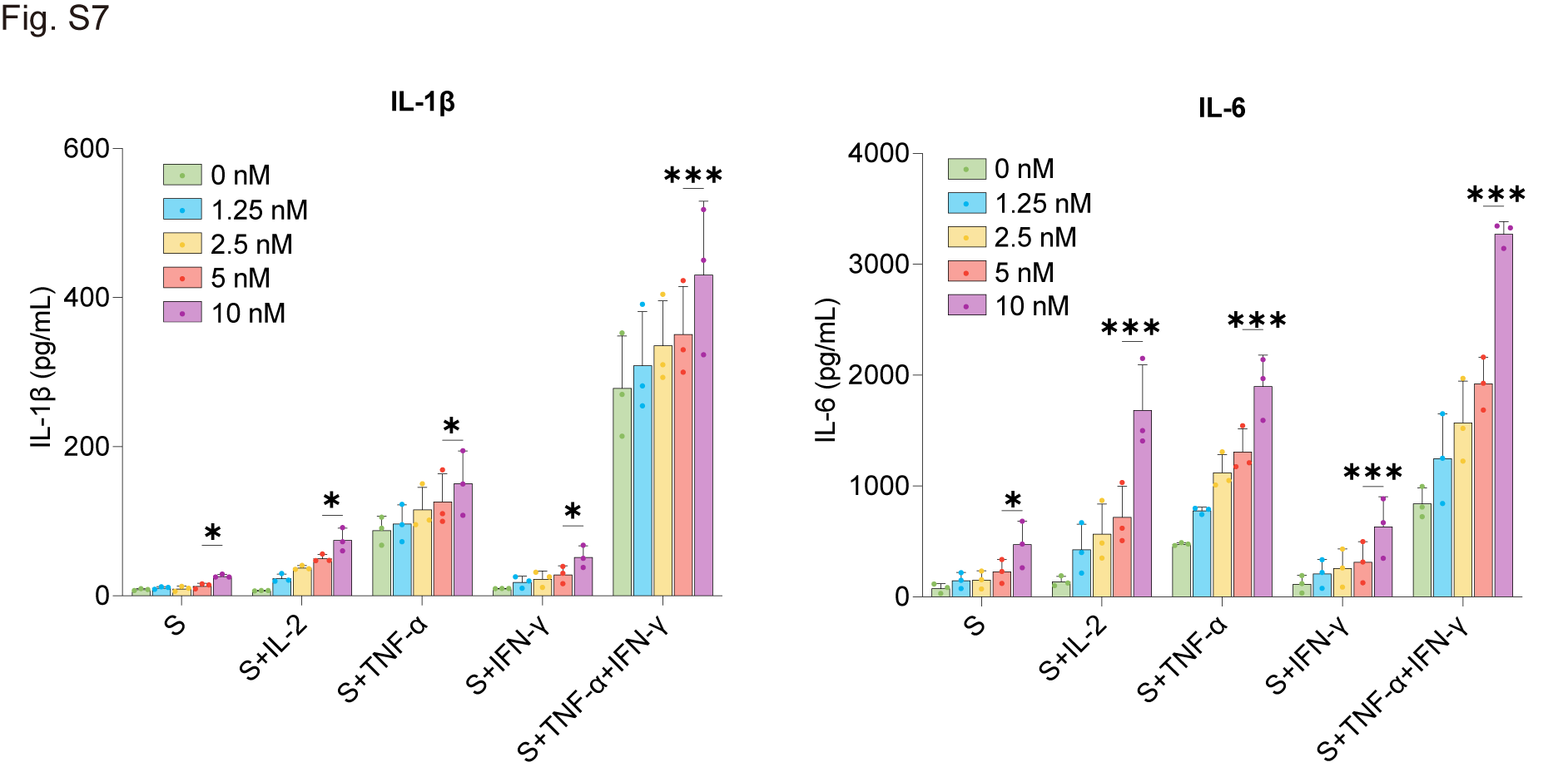

Supplement: Supplementary Figure S7 — The synergistic effects of spike protein with IL-2, TNF-α and IFN-γ were spike protein dose-dependent. Quantifying concentrations of IL-1β and IL-6 in supernatants of PBMCs stimulated by different concentrations of spike protein combining with IL-2, TNF-α and IFN-γ for 16 hrs according to CBA (n = 3 biological replicates). S, spike protein. Data are presented as mean ± SD. *p < 0.05, **p < 0.01, and ***p < 0.001 as analyzed by two-way ANOVA. [file Image7.tif]
